# The Dual Effect of Participation Level on Consumer Participation in Participatory CSR: The Role of CSR Fit and Social Support

**DOI:** 10.3390/bs13040285

**Published:** 2023-03-26

**Authors:** Dongho Yoo

**Affiliations:** Department of Business Administration, Halla University, 28, Halladae-gil, Gangwon, Wonju 26404, Republic of Korea; dongho.yoo@halla.ac.kr

**Keywords:** corporate social responsibility (CSR), participatory CSR, participation level, CSR fit, social support

## Abstract

Corporate social responsibility (CSR) provides companies with two benefits: creating social value and strengthening consumer relationships. Companies implement various types of CSR to maximize the positive effects of CSR, participatory CSR being one of these types. However, although the number of companies using participatory CSR in practice is increasing, academic interest in the effectiveness of participatory CSR has been insufficient. In particular, prior studies on the consumer perception of the participation level presented in participatory CSR do not show clear results. This study examines the influence of the participation level based on CSR fit and social support. The results of this study indicate that when there is a high CSR fit, consumers perceive the participation level as a benefit. However, when the CSR fit is low, consumers perceive the participation level as a cost. Additionally, the results show that the interactive effect of the participation level and CSR fit occurs only when there is less social support. When there is strong social support, consumers perceive the participation level as a benefit regardless of the CSR fit. Finally, the academic and practical implications of the results of this study are presented.

## 1. Introduction

Corporate social responsibility (CSR) plays the most important role in corporate sustainable management [1]. The social demand for corporate social value creation is continuously increasing, and companies are responding to this through CSR activities [2]. Companies are using CSR activities to manage relationships with stakeholders beyond contributing to society. In other words, CSR is a strategic tool that not only creates social value for companies but also positively improves the corporate image and strengthens relationships with consumers [3].

Companies have developed and implemented various types of CSR to effectively achieve the two goals of creating social value and improving consumer relationships [4]. Of these types, participatory CSR is a strategy used to increase the effectiveness of CSR through interaction with consumers [5,6]. By inducing consumer participation in CSR activities, companies enable consumers to experience identification and ensure they feel the sincerity of these activities [6,7]. As a result, participatory CSR can lead to positive evaluations and behavioral intentions of consumers [8]. For example, Coca-Cola implemented participatory CSR by donating money based on the consumers’ participation in recycling, leading to a positive evaluation by consumers [9]. Furthermore, many companies are conducting CSR activities by donating a portion of a product’s revenue based on consumer purchasing behavior [3]. Companies’ interest in, and practical application of, participatory CSR is steadily increasing, and companies implementing participatory CSR are encouraging consumer participation in a variety of ways. These participation methods include not only relatively easy participation, such as Charity Miles, where donations automatically accumulate while users run, walk, jog, bike, etc., simply by using the mobile app but also participation that requires greater effort, such as Coca-Cola’s campaign where participants must go to a vending machine to recycle. However, previous studies have shown little interest in the level of effort of participation that companies should present to consumers; thus, the results are unclear [4]. According to previous studies, the participation effort required in CSR activities may be perceived by consumers as providing benefits, such as the feeling of a ‘warm glow’ but may also be perceived as causing monetary/non-monetary costs [10]. In other words, consumer participation may negatively impact consumers’ perceptions of CSR. In this regard, this study suggests that the participation level presented in participatory CSR can be perceived by consumers by the opposing concepts of benefits and costs, and examines whether such consumer perception is determined by CSR fit. CSR fit, which means the similarity between the corporate image and a CSR activity domain, is known to affect consumers’ perceptions of benefits/costs and consumer attribution [8,11,12]. According to previous studies, a high level of CSR fit causes consumers to think of corporate CSR motives as a method of social value creation and focuses on the purpose of CSR, but a low level of CSR fit results in them thinking of CSR motives as a method of corporate profit and focuses on the costs produced by CSR [8,12]. Based on this, this study predicted that the level of participation presented in participatory CSR would be perceived as a benefit to consumers when CSR fit is high, while the participation level would be perceived as a cost when CSR fit is low. Furthermore, this study investigated ways to reduce consumers’ perception of cost and increase their intention to participate in CSR in order to provide richer practical implications to companies. Based on a previous study that determines that social support reduces the importance of cost [13], we examined whether the perceived costs of consumers according to the participation level presented in participatory CSR are alleviated by social support. This is expected to suggest effective ways to implement participatory CSR in companies.

## 2. Theoretical Background

### 2.1. Participatory CSR

CSR refers to maximizing a company’s positive influence on society and minimizing its negative influence to create social value [14]. As the social demand for the sustainable management of companies increases, most companies are engaging in CSR activities in response to this, and CSR has become the most important factor in the sustainable management of a company [1]. CSR activities are helpful for companies in various ways, as they not only increase the social value of a company, they also help strengthen relationships with stakeholders, including consumers [7,15,16]. Indeed, many studies have shown that CSR helps to improve a company’s image and has a positive effect on consumers’ attitudes towards products and purchase intentions [1,17,18,19,20]. Thus, CSR is an effective strategic tool for businesses to meet social needs and improve relationships with consumers [3,4]; thus, CSR serves as a link between consumers and businesses.

Companies are now strategically developing and implementing various types of CSR to maximize its positive effects [4]. The most representative example is participatory CSR. Participatory CSR refers to CSR activities in which a company’s CSR activities are implemented through the participation of consumers [21,22]. Consumer participation in CSR allows consumers to positively perceive corporate CSR motives and increases their awareness of authenticity [5,6]. In addition, participatory CSR enables consumers to experience identification with CSR activities, thereby bringing about a positive attitude toward the company [7]. In other words, participatory CSR reduces consumers’ doubts about a company’s CSR activities and leads to a beneficial attitude towards the company [8].

A distinctive feature of participatory CSR is that the participation of consumers determines the success of CSR [4]. Accordingly, companies have been encouraging consumers to participate in various ways. For example, Coca-Cola conducted participatory CSR using reverse vending machines (RVM). In this campaign, when consumers recycled plastic cups through RVM, Coca-Cola accumulated points that reflect the number of recycled cups and donated this amount of money to support the Olympic Games. In addition, the Seoul Transportation Corporation in Korea conducted a CSR campaign in which donations that reflected the number of stairs that consumers walked at subway stations were accumulated [4]. Participation in participatory CSR includes financial, as well as non-monetary participation related to consumer behavior. A representative participatory CSR that asks consumers for financial participation is known as cause-related marketing (CRM). CRM refers to CSR in the form of consumers purchasing a product and having a portion of the product revenue donated to solve social problems [23]. In other words, consumers participate in CSR by purchasing products, and companies donate to social agendas based on consumer participation. In participatory CSR, companies can set different levels of participation even if the form of participation required by consumers is the same. For example, P&G set the participation level of CRM to be a 1% donation of product revenue, while Tommy Hilfiger set the participation level to a 50% donation. However, although companies are encouraging consumers to participate in CSR in various ways, previous studies have been insufficient in examining how consumers interpret the company’s request for participation. For this reason, the results of previous studies are unclear regarding what a company should set the level of participation to [4,5]. While some studies suggest that the level of participation presented in CSR has a positive effect on consumer response [24,25,26], other studies have shown a negative effect [27]. Therefore, understanding how consumers perceive the level of participation in CSR is important for the success of participatory CSR.

In this regard, recent prior studies have shown that the level of participation presented in CSR can be interpreted by two opposing roles for consumers. Yoo, Kim and Doh [3] showed that participatory effort in monetary form can be perceived as a benefit to consumers, but it can also be perceived as a monetary sacrifice. According to their experimental results, consumers who perceive their participation effort as a benefit have a positive intention to participate as their participation effort requested by the company increases, whereas those who perceived participation effort as a monetary sacrifice have a negative intention to participate as the participation effort requested by the company increases. In a similar vein, Ahn and Lee [5] revealed that a non-monetary form of participation effort may be perceived by consumers through the positive concept of a ‘warm glow’, or the negative concept of cost. Howie, Yang, Vitell, Bush and Vorhies [10] suggested that the level of participation required in CSR can be perceived by consumers as being characterized by the opposing concepts of benefits and costs. That is, the participatory effort presented to consumers in participatory CSR may be perceived as a benefit to consumers but may also be recognized as a monetary/non-monetary cost. If consumers perceive the benefits more than the costs, they have an intention to participate in the CSR campaign, but if they perceive the costs more than the benefits, their intention to participate becomes negative. Therefore, it is important for the success of participatory CSR to examine what consumers perceive as a participatory effort of participatory CSR and what factors determine it. In this study, this is examined through the CSR fit.

### 2.2. CSR Fit

Fit is defined as the perceived similarity between two concepts, such as a company’s product, brand image, or target market [23]. Therefore, the CSR fit refers to the perceived similarity between the characteristics of a company and the characteristics of its CSR activities [11]. 

The CSR fit influences the consumer’s evaluation of CSR activities and companies and affects their purchasing behavior [28]. Previous studies have shown that the higher the CSR fit, the more positive the evaluation of consumers. Speed and Thompson [29] found that a high CSR fit stimulates consumers’ interest and increases their liking of a company. Becker-Olsen, Cudmore and Hill [11] showed that the higher the CSR fit, the more easily the positive image of CSR activities is transferred to the company. They also argued that a low CSR fit increases consumers’ thinking, making them question the CSR motives of companies. In other words, CSR fit influences the way consumers think about corporate CSR motives.

This study focuses on consumers’ perceptions of CSR activities based on the CSR fit. According to previous studies, the CSR fit affects consumers’ perceptions of the motives of the companies that implement CSR [11]. When there is a high CSR fit, consumers think that the motive of the company is to create social value, and thus, they have a positive attitude toward the company and the CSR activities, whereas when the CSR fit is low, they perceive the motive as a pursuit of profit and have a negative attitude [8]. The CSR fit also affects consumers’ perceptions of benefits and costs. This is because the consumers’ perceptions of the motives of companies implementing CSR influence their perceptions of benefits/costs. Indeed, Habel, Schons, Alavi and Wieseke [12] showed that consumer perceptions of business motives influence their price perceptions. Specifically, consumers who judged the corporate motive for CSR activities as altruistic perceived the price as a benefit, but those who judged the corporate motive as self-serving perceived the price as a cost. When consumers make internal attributions to a company’s CSR activities, altruistic and social awareness is activated, so they perceive the price as a benefit to society. However, when they make external attributions, the perception of cost is activated and they perceive the price as a cost [11,30]. 

Applying this to my research, I expect that the way consumers perceive the participation level presented in participatory CSR will vary depending on the CSR fit. When there is a high CSR fit, consumers will focus on the size of benefits, which will vary according to the level of participation. Therefore, I predict that as the level of participation increases, consumers will have a positive intention to participate in CSR by judging that the benefits of CSR increase. However, when the CSR fit is low, consumers will focus on the costs incurred by their CSR participation. Due to this, I expect that consumers will have a negative intention to participate in CSR, judging that the cost increases as the level of participation increases.

### 2.3. Social Support

Social support refers to the attention and support received from others in social relationships [31]. Consumers who receive social support feel that they are cared for, encouraged, valued, and guided by others [32,33]. Social support has long been considered a part of people’s psychological defense mechanisms and has played a role in enhancing self-esteem in social relationships [13,34].

In this study, it was expected that social support would affect the effectiveness of participatory CSR for two reasons. First, social support activates consumers’ social motivation [35,36]. Social motivation is a representative antecedent factor that leads consumers to engage in pro-social behavior [37]. That is, social support induces the pro-social behavior of consumers through the activation of social motivation. Social motivation also drives consumers to pursue social goals [35,36]. This means that social support induces consumers to focus on the purpose and value of corporate pro-social activities such as CSR. Second, social support can reduce consumers’ desire for money [13]. According to previous studies, social support can reduce the importance of money by activating consumers’ altruistic motives while simultaneously satisfying their needs for social relationships [13,38]. This is because the need for social relationships and the need for money have an interchangeable effect [39]. In other words, the need for money may reduce the need for social relationships [40,41], but satisfying the need for social relationships also reduces the need for money [42,43]. Indeed, Rindfleisch et al. [44] showed that family resources, such as guidance and emotional support, reduced the materialism of family members. Xu, Zhou, Ye and Zhou [13] demonstrated that social support reduces the spending pain experienced by consumers when purchasing a product, as social support decreases consumers’ awareness of the importance of money. Furthermore, Lasaleta et al. [45] showed that nostalgia that satisfies consumers’ social relationship needs reduces the importance of money.

In summary, social support drives consumers to pursue social goals and decreases their perception of costs. Applying this to the context of participatory CSR, it is expected that the interaction effect of the participation level and CSR fit on perceived benefits and costs will be different due to social support. First of all, if social support is not activated, the influence of the participation level on consumer perception will depend on the CSR fit. As mentioned earlier, when there is a high CSR fit, consumers perceive the level of participation as the size of the benefit. As a result, they have a positive intention to participate in CSR, judging that the benefits increase as the level of participation increases. However, when the CSR fit is low, the level of participation is perceived as the size of the cost, judging that the higher the level of participation, the higher the cost, resulting in a negative intention to participate in CSR. Based on the above discussion, the following hypotheses were derived.

**Hypothesis 1 (H1).**  *In the group in which social support is not activated, the influence of the level of participation on consumers’ responses (perceived benefits, perceived costs, and participation intention) is changed by the CSR fit.*

**Hypothesis 1a (H1a).**  *When the CSR fit is high, consumers perceive more benefits at the high level of participation than at the low level of participation. However, when the CSR fit is low, the level of participation does not significantly affect the perceived benefit.*

**Hypothesis 1b (H1b).**  *When the CSR fit is high, the level of participation does not significantly affect the perceived costs. However, when the CSR fit is low, consumers perceive more costs at a high level of participation than at a low level of participation.*

**Hypothesis 1c (H1c).**  *When there is a high CSR fit, consumers have a more positive participation intention at the high level of participation than at the low level of participation, whereas when the CSR fit is low, they have a more negative participation intention at the high level of participation than at the low level of participation.*

However, if social support is activated, it is expected that the interaction effect of the participation level and CSR fit on consumer perception will disappear. Social support drives consumers to pursue social goals [35,36]. Social support also reduces consumers’ perceptions of cost, making them focus on the value derived from CSR activities [3,13]. Therefore, when social support is activated, consumers perceive more benefits than costs as their participation level increases, regardless of CSR fit, and have a positive intention to participate. In other words, the interaction effect of the participation level and CSR fit disappears. Through this, the following hypotheses were derived.

**Hypothesis 2 (H2).**  *When social support is strong, the interaction effect of the CSR fit and participation level on: (a) Perceived benefits; (b) Perceived costs; and (C) Participation intention, is diluted.*

**Hypothesis 3 (H3).**  *The interaction effect of the participation level, CSR fit, and social support on the participation intention is mediated by the perceived benefits and costs.*

## 3. Materials and Methods

### 3.1. Data Collection and Sample

**Figure 1:**
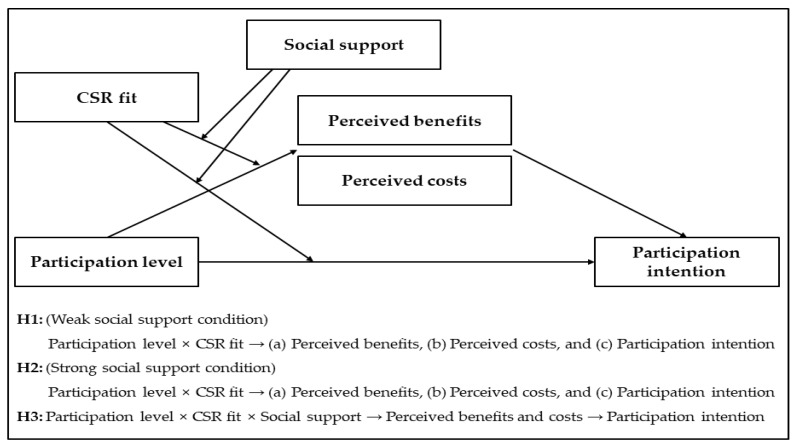
Conceptual model.

To test the hypothesis of this study, the following experiment was conducted. A 2 (participation effort: low vs. high) × 2 (CSR fit: low vs. high) × 2 (social support: weak vs. strong) between-subjects design was applied. Figure 1 presents the framework of this study. 

A total of 333 American participants were recruited as sample for the experiment via the Amazon Mechanical Turk (Mturk). The survey of this study was conducted between 13 March 2022 and 23 March 2022. Each participant was randomly assigned to one of eight groups according to three variables. A total of 59.16% (*n* = 197) of them were male, and their average age was 37.78 (SD = 9.91, age range: 21–73) (see Table 1). The sample size of each group was 39–44 people.

### 3.2. Experimental Stimlus

In this study, McDonald’s was selected as the company to be presented to the subjects. McDonald’s is one of the most used global companies by consumers and has been creating social value through various types of CSR activities for a long time [46]. Moreover, McDonald’s has been used as a brand for experiments in various studies related to consumer behavior [47,48]. The experimental stimulus was produced in the form of a scenario and consisted of the participatory CSR content that McDonald’s was implementing. The participatory CSR presented in the scenario was implemented using cause-related marketing. Cause-related marketing refers to marketing activities in which a portion of the product revenue is donated to support a social cause when consumers purchase a product [49], and is a representative type of participatory CSR. A pretest was conducted using 40 subjects recruited through Amazon Mturk to determine the CSR fit and level of donation. To select the area of the CSR activity to be used in the experiment, five types of CSR activities used in the study of Yoo and Lee [1] were presented to the subjects, and they were then asked to respond to the fit with McDonald’s. As a result of the pretest, I selected the “Saving starving children” campaign as a CSR activity with a high fit and “Saving polar bears” as a CSR activity with a low fit. Subjects perceived a greater similarity to McDonald’s in the CSR activity of high fit (M = 4.90) than the CSR activity of low fit (M = 2.95; t = 5.137, *p* < 0.001). The level of participation was manipulated to the level of donation presented to consumers in cause-related marketing. According to Folse et al. [50] and Chang [51], a low level of participation is set as a donation of 5% of the product revenue, and a high level of participation is set as a donation of 40% of the product revenue. As a result of the pretest, the subjects responded that more participation effort was needed at the high level of donation (M = 5.00) than at the low level of donation (M = 3.25; t = 3.382, *p* < 0.01). 

### 3.3. Methods

The subjects who participated in the experiment first performed a task related to social support. The subjects’ social support was manipulated in the manner used in the study of Schulz and Decker [52] and Xu, Zhou, Ye and Zhou [13]. In the strong social support group, subjects were asked to recall three people who had provided them with help, support, and guidance in the past and then write their initials. In the weak social support group, subjects were asked to recall three celebrities (such as athletes, singers, movie stars, etc.) and write their initials down. Following this, the subjects responded to the manipulation check items of social support. The manipulation check of social support was measured through the five items used in the study of Xu, Zhou, Ye and Zhou [13].

After the social support task, the subjects were asked to read a scenario consisting of the contents of a company’s CSR activities (see Appendix A). The scenario discussed how McDonald’s is currently executing cause-related marketing, and when consumers purchase McDonald’s products, a portion of the product revenue will be donated to help solve social problems. The level of participation (low, a donation of 5% of product revenue; high, a donation of 40% of product revenue) and CSR fit (low, saving polar bears; high, saving starving children) presented to consumers in this scenario were presented differently depending on the experimental group based on the results of the pretest. After reading the scenario, the subjects responded to the manipulation check items of the CSR fit [1] and participation level [53], brand familiarity [1], perceived benefits and costs, and participation intention. Perceived benefits refer to the social benefits and warm glow that the subjects felt by participating in the CSR campaign and were measured by the four items used by Andrews et al. [54]. Perceived costs are defined as monetary/non-monetary costs recognized when participating in CSR campaigns and were measured by the two items used by Bridger and Wood [55]. Participation intention represents the intention of the subjects to participate in the proposed CSR campaign and was measured by the four items used by Grau and Folse [56]. Brand familiarity was measured to minimize the influence of external variables that may occur as a result of using the actual brand. The reliability of all items was 0.800 or higher (see Table 2).

## 4. Results

### 4.1. Manipulation Checks

Prior to the analysis of the experimental results, a manipulation check was performed to ensure that the experimental stimuli were properly manipulated. The results of the manipulation check show that the experimental stimulus was manipulated successfully.

First, as a result of an ANCOVA of 2 (participation level) × 2 (CSR fit) × 2 (social support) for perceived participation level, the main effect of participation level was significant (F = 61.111, *p* < 0.001). Subjects perceived the level of effort required to engage in CSR as higher when the participation level was high (M = 5.55) than when it was low (M = 4.33). The influence of the other variables was not significant.

As a result of an ANCOVA of 2 (participation level) × 2 (CSR fit) × 2 (social support) for perceived CSR fit, the main effect of the CSR fit was significant (F = 74.859, *p* < 0.001). The subjects perceived the similarity between corporate image and CSR activities as greater when the CSR fit was high (M = 5.65) than when it was low (M = 4.24). The influence of other variables was not significant.

Finally, as a result of an ANCOVA of 2 (participation level) × 2 (CSR fit) × 2 (social support) for perceived social support, the main effect of social support was significant (F = 33.304, *p* < 0.001). Subjects with activated social support (M = 5.54) believed that they were receiving more social support, encouragement, and help than subjects without activation (M = 4.64). The influence of other variables was not significant.

### 4.2. Perceived Benefits

**Figure 2:**
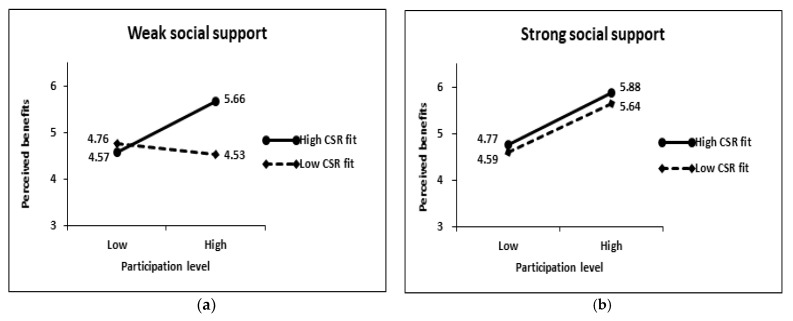
Interaction effects on perceived benefits: (**a**) Weak social support condition; (**b**) Strong social support condition.

Model 12 of the PROCESS macro was performed for the 2 (participation level) × 2 (CSR fit) × 2 (social support) between-subjects analysis for perceived benefits, perceived costs, and participation intentions (H1 and H2) and moderated mediation analysis (H3). The results of the 2 × 2 × 2 between-subjects analysis on perceived benefits revealed that the main effects of participation level (t = 0.36, *p* > 0.1), CSR fit (t = −1.27, *p* > 0.1), and social support (t = −1.41, *p* > 0.1) were not significant (see Table 3 and Table 4). More importantly, the three-way interaction effect of the three variables was significant (t = −2.30, *p* < 0.05). In the case of weak social support, the influence of participation level on perceived benefits was determined by the CSR fit (see Figure 2). Subjects perceived more benefits at high levels of participation (M = 5.66) than at low levels of participation (M = 4.57) when the CSR fit was high (F = 14.865, *p* < 0.001), but when the CSR fit was low, they did not perceive benefits differently depending on the participation level (low: 4.76 vs. high: 4.53; F = 0.537, *p* > 0.1). This supports H1a. However, in the case of strong social support, the influence of the CSR fit on perceived benefits disappeared. Subjects perceived more benefits at high levels of participation than at low levels of participation both when the CSR fit was low (low participation level: 4.59 vs. high participation level: 5.64; F = 11.290, *p* < 0.01) and when it was high (low participation level: 4.77 vs. high participation level: 5.88; F = 11.050, *p* < 0.01). Therefore, H2a was supported.

### 4.3. Perceived Costs

**Figure 3:**
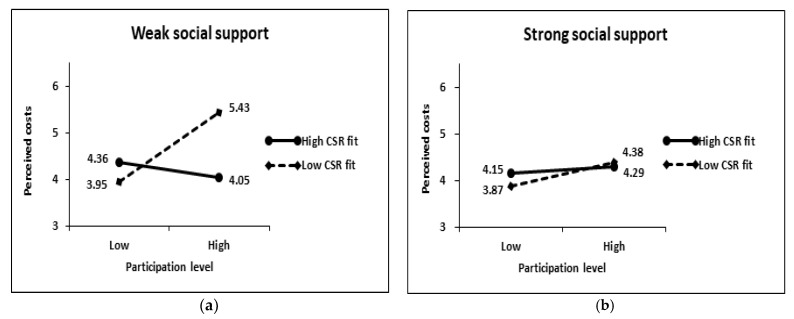
Interaction effects on perceived costs: (**a**) Weak social support condition; (**b**) Strong social support condition.

The results of the 2 × 2 × 2 between-subjects analysis on perceived costs showed that the main effects of participation level (t = 1.09, *p* > 0.1), CSR fit (t = 0.58, *p* > 0.1), and social support (t = 1.37, *p* > 0.1) were not significant (see Table 3 and Table 4). However, the three-way interaction effect of the three variables was significant (t = 2.25, *p* < 0.05). In the case of weak social support, subjects’ perceived costs varied by participation level and CSR fit (see Figure 3). Subjects did not perceive costs differently depending on the participation level when the CSR fit was high (low participation level: 4.36 vs. high participation level: 4.05; F = 1.236, *p* > 0.1), but when the CSR fit was low, they perceived more costs at high levels of participation (M = 5.43) than at low levels of participation (M = 3.95; F = 16.853, *p* < 0.001). Therefore, H1b was supported. However, in the case of strong social support, the influence of the participation level and CSR fit on perceived costs disappeared. Subjects did not perceive cost differently according to the level of participation, both when the CSR fit was low (low participation level: 3.87 vs. high participation level: 4.38; F = 2.001, *p* > 0.1) and when it was high (low participation level: 4.15 vs. high participation level: 4.29; F = 0.256, *p* > 0.1). This means that H2b was supported.

### 4.4. Intention to Participate in CSR

**Figure 4:**
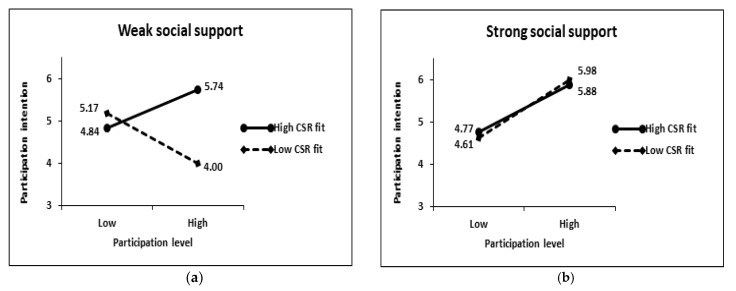
Interaction effects on participation intention: (**a**) Weak social support condition; (**b**) Strong social support condition.

The results of the 2 × 2 × 2 between-subjects analysis for participation intention showed that the main effects of participation level (t = −0.86, *p* > 0.1), CSR fit (t = 1.65, *p* > 0.1), and social support (t = −0.82, *p* > 0.1) were not significant (see Table 3 and Table 4). Additionally, the three-way interaction effect of the three variables was significant (t = −2.80, *p* < 0.01). In the case of a low level of social support, the influence of participation level on participation intention was determined by the CSR fit (see Figure 4). When the CSR fit was high, subjects had more positive participation intentions at the high participation level (M = 5.74) than at the low participation level (M = 4.84; F = 8.925, *p* < 0.01), whereas when the CSR fit was low, they had more positive participation intentions at the low participation level (M = 5.17) than at the high participation level (M = 4.00; F = 13.721, *p* < 0.01). This supports H1c. However, when social support was strong, the influence of the CSR fit on participation intention disappeared. Subjects had more positive participation intentions at high levels of participation than at low levels of participation both when CSR fit was low (low participation level: 4.61 vs. high participation level: 5.98; F = 18.370, *p* < 0.001) and when it was high (low participation level: 4.77 vs. high participation level: 5.88; F = 11.255, *p* < 0.01). Therefore, H2c was supported.

### 4.5. Moderated Mediation Analysis

A Moderated mediation analysis was performed to test Hypothesis 3, which explains the mechanism of this study. For this, Model 12 of the PROCESS macro (bootstrapping analysis using 10,000 resamples) was used [57,58]. The results of analysis by setting participation level as an independent variable, the CSR fit and social support as moderate variables, perceived benefit and cost as mediate variables, and participation intention as a dependent variable showed significant mediating effects of perceived benefits and costs. In other words, the indirect effects of perceived benefits (indirect effect: 1.07, 95% CI: 0.18 to 2.07) and perceived costs (indirect effect: 0.12, 95% CI: 0.02 to 0.32) were significant. Therefore, Hypothesis 3 was supported.

## 5. Discussion and Conclusions

This study was conducted to confirm how the level of participation affects consumers’ perception of participatory CSR and to suggest ways to increase consumer participation. For this, the role of the CSR fit and social support was examined. The results of this study are summarized as follows. First, the level of participation presented in participatory CSR was perceived as providing benefits to consumers, as well as costs. Furthermore, the CSR fit determined this consumer perception. When the CSR fit was high, the level of participation had a positive effect on the intention to participate in the CSR campaign because consumers perceived the level of participation as a benefit. However, when the CSR fit was low, the level of participation had a negative effect on the intention to participate because consumers perceived the level of participation as a cost. Furthermore, this study confirmed that social support alleviates consumers’ perception of costs. In other words, when social support was activated, consumers perceived the level of participation as a benefit regardless of CSR fit. As a result, the higher the level of participation, the more positive consumers’ intention to participate in the CSR campaign were.

This study has the following academic implications. First, this study confirmed that the level of participation presented in participatory CSR can be perceived by consumers as being characterized by the opposing concepts of benefits and costs, and that this consumer perception affects the intention to participate in CSR campaigns. These results are consistent with the results of previous studies [3,4,5] that indicate that consumers’ monetary and non-monetary efforts in participatory CSR can be perceived as opposing concepts by consumers. In other words, this study expanded the results of previous studies. The results of this study are meaningful in that they revealed the mechanism of the participation level in participatory CSR. Second, this study suggests that the influence of the level of participation on consumers’ perception is determined by the CSR fit. Although previous studies have suggested that there is the possibility of consumers’ opposing perceptions of participation levels [3,4,5], there was insufficient consideration of the factors that determine this. This study expands on previous studies by suggesting that the dual roles of benefits and costs of participation levels in participatory CSR are determined by the CSR fit. Additionally, this study revealed that the CSR fit not only affects consumers’ perceptions of authenticity and evaluations but also affects consumer perceptions related to the level of participation. In other words, this study contributed to the expandability of research related to CSR, fit as well as consumer participation. Finally, this study confirmed the role of social support in reducing consumers’ perception of costs, as this expanded on studies on social support by proving that the influence of social support, which decreases the importance of money, also occurs strongly in the context of participatory CSR.

The practical implications of this study are as follows. In participatory CSR, the level of participation is an attribute that corporate marketers can set relatively easily [3]. However, the results of this study show that it is necessary for companies to carefully consider the level of participation because it may be perceived negatively by consumers. Additionally, this study suggests that companies can control the influence of the level of participation on consumers’ perceptions through CSR fit. Specifically, when companies engage in CSR activities with a high fit, it is desirable to set the level of participation high. This is because a high CSR fit allows consumers to perceive the level of participation as a benefit. On the other hand, when the CSR fit is low, companies should set the level of participation relatively low. This is because a low level of CSR fit leads consumers to perceive the level of participation as a cost. Finally, social support reduces consumers’ perceptions of cost. Therefore, companies that want to implement participatory CSR need to contemplate ways to utilize social support.

Although this study has various academic and practical implications, it also has the following limitations. First, the brand used in the experiment of this study was McDonald’s, and in future research, it is necessary to conduct experiments using more diverse brands. This will help generalize the results of this study. In addition, according to previous studies, the image associated with a brand affects the success of CSR. For example, according to a study by Luo and Bhattacharya [59], the better the brand quality as perceived by consumers, the greater the positive effect of CSR. In future research, it is necessary to examine whether brand image affects consumer perception according to the level of participation presented in participatory CSR by using various brands.

Second, in the experiment of this study, participatory CSR was presented to the subjects as cause-related marketing. However, participatory CSR includes many types of CSR activities other than cause-related marketing. For example, Ahn and Lee [5] manipulated the level of engagement with the number of mobile app accesses. Future research can provide richer practical implications to companies by conducting experiments that manipulate more diverse participation methods. Finally, future studies should conduct experiments by manipulating social support in more diverse ways. In other words, in future research, it is necessary to conduct an experiment using a more practical method, such as warm conversations with service workers at the point of purchase [13], as well as the priming method of this study.

## Figures and Tables

**Table 1:** Characteristics of sample.

	Characteristics	N	%
Gender	Male	196	58.9
	Female	137	41.1
Age	20–29	65	19.5
	30–39	139	41.8
	40–49	87	26.1
	50–59	30	9.0
	60–69	10	3.0
	70–79	2	0.6

**Table 2:** Scale items.

Variables	Items	Cronbach’s Alpha
Social support	“There is a special person who is around when I am in need.”	0.943
	“There is a special person with whom I can share my joys and sorrows.”	
	“My friends really try to help me.”	
	“I can count on my friends when things go wrong.”	
	“There is a special person in my life who cares about my feelings.”	
Participation level	“I need to put a lot of effort into participation in this campaign.”	0.909
	“I have to spend a lot of effort in participation in this campaign.”	
	“I feel that I spent a lot of effort in trying to participate in this campaign.”	
CSR fit	“The fit between McDonald’s and the CSR activities is high.”	-
Brand familiarity	“I am familiar with McDonald’s.”	-
Perceived benefits	“I derive benefit from supporting good causes by products of McDonald’s.”	0.893
	“After purchasing products of McDonald’s, I am satisfied as my money helps support a good cause.”	
	“I like that the McDonald’s uses my money to support a good cause.”	
Perceived costs	“I have to spend a lot of resources (in terms of monetary/nonmonetary sacrifice, including time or money) to participate in this campaign.”	0.849
	“Participating in this campaign is very demanding for me (e.g., sacrifice of time or money).”	
Participation intentions	“I think this CRM campaign is a good idea.”	0.937
	“I would be willing to participate in this campaign.”	
	“I would consider participation this campaign.”	
	“It is likely that I would contribute to this cause by getting involved in this campaign.”	

**Table 3:** Results of 2 × 2 × 2 between-subjects analysis using PROCESS Macro Model 12.

Dependent Variables	Perceived Benefits (R^2^ = 0.154)	Perceived Costs (R^2^ = 0.089)	Participation Intention (R^2^ = 0.628)
	t	t	t
Brand familiarity	3.32 **	−2.30 *	−1.34
Perceived benefits (mediator)	-	-	19.24 ***
Perceived costs (mediator)	-	-	−2.32 *
Social support (A)	−1.41	1.37	−0.82
CSR fit (B)	−1.27	0.58	−1.65
Participation level (C)	−0.36	1.09	−0.86
A × B	1.84	−1.46	2.06 *
A × C	1.61	−1.85	1.36
B × C	1.45	−1.06	2.24 *
A × B × C	−2.30 *	−2.25 *	−2.80 **

Note: * *p* < 0.05, ** *p* < 0.01, *** *p* < 0.001.

**Table 4:** Study results: means and standard deviations.

Social Support	CSR Fit	Participation Level	Perceived Benefits	Perceived Costs	Participation Intention
High	High	Low	4.77 (1.62)	4.15 (1.79)	4.77 (1.68)
		High	5.88 (0.79)	4.29 (1.81)	5.88 (0.87)
	Low	Low	4.59 (1.50)	3.87 (1.65)	4.61 (1.66)
		High	5.64 (1.09)	4.38 (1.65)	5.98 (0.79)
Low	High	Low	4.57 (1.71)	4.36 (1.93)	4.84 (1.84)
		High	5.66 (0.91)	4.05 (1.84)	5.74 (0.85)
	Low	Low	4.76 (1.69)	3.95 (1.52)	5.17 (1.40)
		High	4.53 (1.84)	5.43 (1.07)	4.00 (2.02)

Note: The numbers in parentheses represent standard deviations.

**Figure A1:**
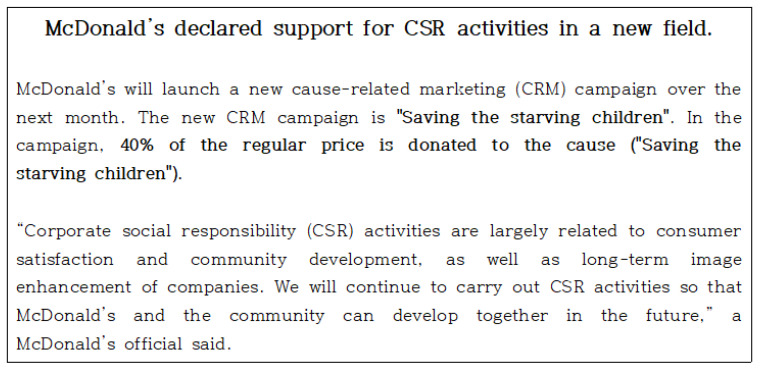
High CSR fit and high participation level condition.

**Figure A2:**
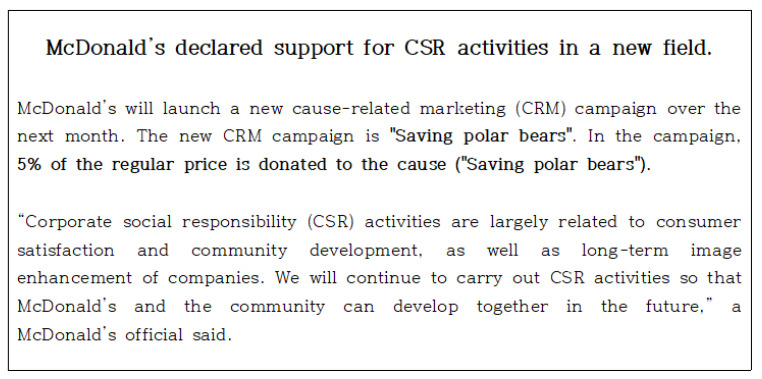
Low CSR fit and low participation level condition.

## Data Availability

The datasets analyzed during the study are available on request from corresponding author.

## References

[1] Yoo D., Lee J. (2018). The effects of corporate social responsibility (CSR) fit and CSR consistency on company evaluation: The role of CSR support. Sustainability.

[2] Du S., Bhattacharya C.B., Sen S. (2010). Maximizing business returns to corporate social responsibility (CSR): The role of CSR communication. Int. J. Manag. Rev..

[3] Yoo D., Kim J., Doh S.-J. (2018). The dual processing of donation size in cause-related marketing (CRM): The moderating roles of construal level and emoticons. Sustainability.

[4] Ahn Y., Lee J. (2021). The role of anthropomorphic messengers in sustainable participatory corporate social responsibility: Focusing on messenger’s facial expression and participation effort. Sustainability.

[5] Ahn Y., Lee J. (2020). The effect of participation effort on CSR participation intention: The moderating role of construal level on consumer perception of warm glow and perceived costs. Sustainability.

[6] Jarvis W., Ouschan R., Burton H.J., Soutar G., O’Brien I.M. (2017). Customer engagement in CSR: A utility theory model with moderating variables. J. Serv. Theory Pract..

[7] Beise-Zee R. (2011). Corporate social responsibility or cause-related marketing? The role of cause specificity of CSR. J. Consum. Mark..

[8] Kuo A., Rice D.H. (2015). The impact of perceptual congruence on the effectiveness of cause-related marketing campaigns. J. Consum. Psychol..

[9] Company T.C.-C. Coke Piloting Kiosks that Reward Recycling with Charitable Donations. https://www.coca-colacompany.com/news/coke-piloting-kiosks-that-reward-recycling.

[10] Howie K.M., Yang L., Vitell S.J., Bush V., Vorhies D. (2018). Consumer participation in cause-related marketing: An examination of effort demands and defensive denial. J. Bus. Ethics.

[11] Becker-Olsen K.L., Cudmore B.A., Hill R.P. (2006). The impact of perceived corporate social responsibility on consumer behavior. J. Bus. Res..

[12] Habel J., Schons L.M., Alavi S., Wieseke J. (2016). Warm glow or extra charge? The ambivalent effect of corporate social responsibility activities on customers’ perceived price fairness. J. Mark..

[13] Xu Q., Zhou Y., Ye M., Zhou X. (2015). Perceived social support reduces the pain of spending money. J. Consum. Psychol..

[14] Mohr L.A., Webb D.J., Harris K.E. (2001). Do consumers expect companies to be socially responsible? The impact of corporate social responsibility on buying behavior. J. Consum. Aff..

[15] Chu S.-C., Chen H.-T. (2019). Impact of consumers’ corporate social responsibility-related activities in social media on brand attitude, electronic word-of-mouth intention, and purchase intention: A study of Chinese consumer behavior. J. Consum. Behav..

[16] Yoon Y., Gürhan-Canli Z., Schwarz N. (2006). The effect of corporate social responsibility (CSR) activities on companies with bad reputations. J. Consum. Psychol..

[17] Barone M.J., Miyazaki A.D., Taylor K.A. (2000). The influence of cause-related marketing on consumer choice: Does one good turn deserve another?. J. Acad. Mark. Sci..

[18] Brown T.J., Dacin P.A. (1997). The company and the product: Corporate associations and consumer product responses. J. Mark..

[19] Du S., Bhattacharya C.B., Sen S. (2011). Corporate social responsibility and competitive advantage: Overcoming the trust barrier. Manag. Sci..

[20] Sen S., Bhattacharya C.B. (2001). Does doing good always lead to doing better? Consumer reactions to corporate social responsibility. J. Mark. Res..

[21] Abbas M., Gao Y., Shah S.S.H. (2018). CSR and customer outcomes: The mediating role of customer engagement. Sustainability.

[22] Brodie R.J., Hollebeek L.D., Jurić B., Ilić A. (2011). Customer engagement: Conceptual domain, fundamental propositions, and implications for research. J. Serv. Res..

[23] Varadarajan P.R., Menon A. (1988). Cause-related marketing: A coalignment of marketing strategy and corporate philanthropy. J. Mark..

[24] Dahl D.W., Lavack A.M. (1995). Cause-related marketing: Impact of size of corporate donation and size of cause-related promotion on consumer perceptions and participation. 1995 AMA Winter Educators’ Conference Proceedings.

[25] Holmes J.H., Kilbane C. (1993). Cause-related marketing: Selected effects of price and charitable donations. J. Nonprofit Public Sect. Mark..

[26] Moosmayer D.C., Fuljahn A. (2010). Consumer perceptions of cause related marketing campaigns. J. Consum. Mark..

[27] Strahilevitz M. (1999). The effects of product type and donation magnitude on willingness to pay more for a charity-linked brand. J. Consum. Psychol..

[28] De Jong M.D.T., van der Meer M. (2017). How does it fit? Exploring the congruence between organizations and their corporate social responsibility (CSR) activities. J. Bus. Ethics.

[29] Speed R., Thompson P. (2000). Determinants of sports sponsorship response. J. Acad. Mark. Sci..

[30] Foreh M.R., Grier S. (2003). When is honesty the best policy? The effect of stated company intent on consumer skepticism. J. Consum. Psychol..

[31] Mai Y., Wu Y.J., Huang Y. (2021). What type of social support is important for student resilience during COVID-19? A latent profile analysis. Front. Psychol..

[32] Gottlieb B.H., Bergen A.E. (2010). Social support concepts and measures. J. Psychosom. Res..

[33] Minghui L., Lei H., Xiaomeng C., Potměšilc M. (2018). Teacher efficacy, work engagement, and social support among Chinese special education school teachers. Front. Psychol..

[34] Teoh A.N., Chia M.S.C., Mohanraj V. (2009). The comparison between active and passive types of social support: The emotional responses. J. Appl. Biobehav. Res..

[35] Phillips B.N., Iwanaga K., Rumrill S., Reyes A., Wu J.-R., Fleming A.R., Chan F. (2021). Development and validation of the social motivation scale in people with disabilities. Rehabil. Psychol..

[36] Wentzel K.R., Battle A., Russell S.L., Looney L.B. (2010). Social supports from teachers and peers as predictors of academic and social motivation. Contemp. Educ. Psychol..

[37] You S., Lee J., Lee Y. (2022). Relationships between gratitude, social support, and prosocial and problem behaviors. Curr. Psychol..

[38] Ballantine P.W., Stephenson R.J. (2011). Help me, I’m fat! Social support in online weight loss networks. J. Consum. Behav..

[39] Zhou X., Gao D.-G. (2008). Social support and money as pain management mechanisms. Psychol. Inq..

[40] Lane R. (2000). The Loss of Happiness in Market Democracies New Haven.

[41] Vohs K.D., Mead N.L., Goode M.R. (2006). The psychological consequences of money. Science.

[42] Chaplin L.N., John D.R. (2007). Growing up in a material world: Age differences in materialism in children and adolescents. J. Consum. Res..

[43] Pieters R. (2013). Bidirectional dynamics of materialism and loneliness: Not just a vicious cycle. J. Consum. Res..

[44] Rindfleisch A., Burroughs J.E., Denton F. (1997). Family structure, materialism, and compulsive consumption. J. Consum. Res..

[45] Lasaleta J.D., Sedikides C., Vohs K.D. (2014). Nostalgia weakens the desire for money. J. Consum. Res..

[46] Chen X., Huang R., Yang Z., Dube L. (2018). CSR types and the moderating role of corporate competence. Eur. J. Mark..

[47] Monga A.B., John D.R. (2007). Cultural differences in brand extension evaluation: The influence of analytic versus holistic thinking. J. Consum. Res..

[48] Woolley K., Liu P. (2021). How you estimate calories matters: Calorie estimation reversals. J. Consum. Res..

[49] Kotler P., Lee N. (2008). Corporate Social Responsibility: Doing the Most Good for Your Company and Your Cause.

[50] Folse J.A.G., Niedrich R.W., Grau S.L. (2010). Cause-relating marketing: The effects of purchase quantity and firm donation amount on consumer inferences and participation intentions. J. Retail..

[51] Chang C.T. (2008). To donate or not to donate? Product characteristics and framing effects of cause-related marketing on consumer purchase behavior. Psychol. Mark..

[52] Schulz R., Decker S. (1985). Long-term adjustment to physical disability: The role of social support, perceived control, and self-blame. J. Pers. Soc. Psychol..

[53] Xia L., Kukar-Kinney M., Monroe K.B. (2010). Effects of consumers’ efforts on price and promotion fairness perceptions. J. Retail..

[54] Andrews M., Luo X., Fang Z., Aspara J. (2014). Cause marketing effectiveness and the moderating role of price discounts. J. Mark..

[55] Bridger E.K., Wood A. (2017). Gratitude mediates consumer responses to marketing communications. Eur. J. Mark..

[56] Grau S.L., Folse J.A.G. (2007). Cause-related marketing (CRM): The influence of donation proximity and message-framing cues on the less-involved consumer. J. Advert..

[57] Preacher K.J., Hayes A.F. (2008). Asymptotic and resampling strategies for assessing and comparing indirect effects in multiple mediator models. Behav. Res. Methods.

[58] Zhao X., Lynch J.G., Chen Q. (2010). Reconsidering Baron and Kenny: Myths and truths about mediation analysis. J. Consum. Res..

[59] Luo X., Bhattacharya C.B. (2006). Corporate social responsibility, customer satisfaction, and market value. J. Mark..

